# Analyzing how the components of the SOFA score change over time in their contribution to mortality

**DOI:** 10.62675/2965-2774.20240030-en

**Published:** 2024-10-31

**Authors:** Barbara D. Lam, Tristan Struja, Yanran Li, João Matos, Ziyue Chen, Xiaoli Liu, Leo Anthony Celi, Yugang Jia, Jesse Raffa

**Affiliations:** 1 Beth Israel Deaconess Medical Center Department of Medicine Boston MA United States Department of Medicine, Beth Israel Deaconess Medical Center - Boston (MA), United States.; 2 Institute for Medical Engineering and Science Massachusetts Institute of Technology Laboratory for Computational Physiology Cambridge MA United States Laboratory for Computational Physiology, Institute for Medical Engineering and Science, Massachusetts Institute of Technology - Cambridge (MA), United States.; 3 Harvard T.H. Chan School of Public Health Boston MA United States Harvard T.H. Chan School of Public Health - Boston (MA), United States.; 4 Genome Institute of Singapore Agency for Science, Technology and Research Singapore Genome Institute of Singapore, Agency for Science, Technology and Research – Singapore.

**Keywords:** Organ dysfunction scores, Patient discharge, Logistic models, Liver, Central nervous systems, Mortality, Hospitals, Intensive care units

## Abstract

**Objective:**

Determine how each organ component of the SOFA score differs in its contribution to mortality risk and how that contribution may change over time.

**Methods:**

We performed multivariate logistic regression analysis to assess the contribution of each organ component to mortality risk on Days 1 and 7 of an intensive care unit stay. We used data from two publicly available datasets, eICU Collaborative Research Database (eICU-CRD) (208 hospitals) and Medical Information Mart for Intensive Care IV (MIMIC-IV) (1 hospital). The odds ratio of each SOFA component that contributed to mortality was calculated. Mortality was defined as death either in the intensive care unit or within 72 hours of discharge from the intensive care unit.

**Results:**

A total of 7,871 intensive care unit stays from eICU-CRD and 4,926 intensive care unit stays from MIMIC-IV were included. Liver dysfunction was most predictive of mortality on Day 1 in both cohorts (OR 1.3; 95%CI 1.2 - 1.4; OR 1.3; 95%CI 1.2 - 1.4, respectively). In the eICU-CRD cohort, central nervous system dysfunction was most predictive of mortality on Day 7 (OR 1.4; 95%CI 1.4 - 1.5). In the MIMIC-IV cohort, respiratory dysfunction (OR 1.4; 95%CI 1.3 - 1.5) and cardiovascular dysfunction (OR 1.4; 95%CI 1.3 - 1.5) were most predictive of mortality on Day 7.

**Conclusion:**

The SOFA score may be an oversimplification of how dysfunction of different organ systems contributes to mortality over time. Further research at a more granular timescale is needed to explore how the SOFA score can evolve and be ameliorated.

## INTRODUCTION

The importance of the Sequential Organ Failure Assessment (SOFA) score was emphasized by its inclusion in the 2016 Sepsis-3 guidelines as part of the criteria to identify patients with sepsis.^(1)^ A higher SOFA score, the maximum SOFA score, and the pattern of its change have been shown to be reliable predictors of mortality.^(2-5)^ There have been significant advances in critical care medicine since the SOFA score was established over 25 years ago however, and experts are now calling for the score to be reevaluated and modernized.^(6)^

Historically, the six organ components of the SOFA score - respiratory, hematologic, renal, hepatic, neurologic, and cardiovascular - were equally weighted. More recently, researchers have hypothesized that specific organ systems may play a greater role than others do, with some findings suggesting that the Glasgow coma scale (GCS) score, a reflection of neurologic dysfunction, is the most important predictor of mortality.^(7-10)^ These prior studies used single-center cohorts and evaluated how dysfunction of each organ component at one point in time contributed to mortality.

In this study, we aimed to expand on previous work by determining how each organ component of the SOFA score differs in its contribution to mortality risk and how that contribution may change over time.

## METHODS

### Study design

We calculated the SOFA score for each organ component at 24 hours (termed "Day 1") and 168 hours (termed "Day 7") and evaluated how each organ component's score contributed to mortality. The analysis was first performed on a cohort of patients from one tertiary care center and then replicated on a cohort of patients from a multicenter database for comparison.

### Data source

We used two intensive care unit (ICU) datasets to conduct our retrospective cohort study: the Medical Information Mart for Intensive Care IV (MIMIC-IV) database^(11)^ and the eICU Collaborative Research Database (eICU-CRD).^(12)^ The MIMIC-IV is a publicly available database that contains information from real ICU stays of patients admitted to one tertiary academic medical center, Beth Israel Deaconess Medical Center (BIDMC), in Boston, United States, between 2008 and 2019. The data in MIMIC-IV has been previously de-identified, and the institutional review boards of the Massachusetts Institute of Technology (No. 0403000206) and BIDMC (2001-P-001699/14) have both approved the use of the database for research. The eICU-CRD database is a publicly available, multi-center database sourced from the Philips Healthcare eICU Telehealth Program and contains information from over 200,000 admissions to ICUs monitored by eICU-CRD programs across the United States. The eICU-CRD data do not encompass MIMIC-IV data. The use of the eICU-CRD database is exempt from institutional review board approval because of the retrospective design, lack of direct patient intervention, and security schema, for which the reidentification risk was certified as meeting safe harbor standards by an independent privacy expert (Privacert, Cambridge, MA; Health Insurance Portability and Accountability Act Certification No. 1031219-2). Both databases contain comprehensive information from ICU stays, including vital signs, laboratory measurements, medications, and mortality data.

### Organ dysfunction definitions

The SOFA score consists of six organ components: the central nervous system (CNS), respiration, coagulation, liver, renal, and cardiovascular systems. Each component is scored on a scale from 0 to 4, where 0 indicates no organ dysfunction and scores of 1 through 4 indicate worsening organ dysfunction.

We used platelet count to define coagulation abnormalities, bilirubin level to define liver abnormalities, creatinine level to define renal abnormalities, mean arterial pressure (MAP) and/or the dose of vasopressors to define cardiovascular abnormalities, and PaO2/FiO2 to define respiratory abnormalities. The GCS is used for the CNS component. Given that our dataset includes hundreds of hospitals and that the GCS is known to be heterogeneously and inaccurately measured, particularly across different institutions,^(13)^ we imputed a GCS score of 15 for all patients who were intubated or sedated, in line with prior strategies outlined in this field of research.^(14,15)^

### Statistical analysis

We performed a multivariate logistic regression analysis to assess the contribution of each organ component to mortality on Days 1 and 7. We adjusted for potential confounders, including age, sex, race, and baseline health status, via the Charlson comorbidity index (CCI). The odds ratios (ORs) with 95% confidence intervals (95%CIs) were calculated. We then performed a sensitivity analysis to assess how the presence of patients with common chronic diseases involving different organ components may affect the results. We removed patients with congestive heart failure (CHF), cirrhosis, chronic obstructive pulmonary disease (COPD), asthma, and chronic kidney disease (CKD) stage III or worse and reanalyzed the contributions of each relevant organ component to mortality.

**Figure 1:**
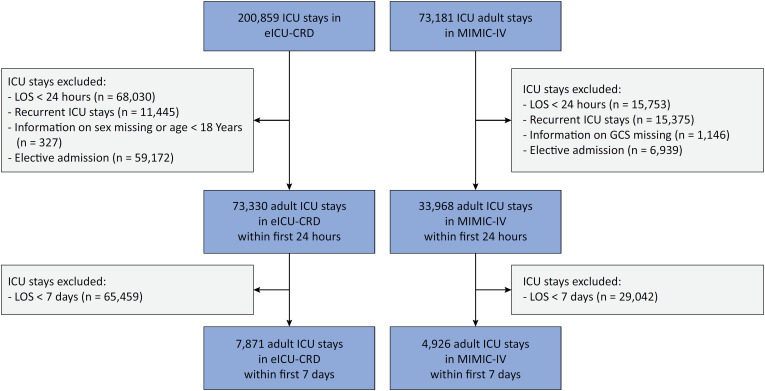
Cohort selection from the eICU Collaborative Research Database and Medical Information Mart for Intensive Care IV databases.

Prior studies in this field of research have used a binary scoring system where a SOFA score of 0, 1, or 2 represents freedom from organ failure, whereas a score of 3 or 4 represents organ failure.^(8,9,16)^ We initially conducted an analysis on the basis of this binary scoring system, and the detailed results are presented in figure 1S and tables 1S - 3S (Supplementary Material). However, to better include the effects of mild organ dysfunction, we performed another analysis using discrete SOFA variables (0, 1, 2, 3, 4) and presented those findings below as well.

### Cohort selection

We included patients 18 years or older admitted to the ICU for at least 168 hours (7 days) whose demographic and SOFA score data were available. If patients were admitted to the ICU multiple times, we only included the first stay that met the inclusion criteria. Elective admissions were excluded by identifying International Classification of Diseases (ICD) codes associated with an elective stay in the MIMIC-IV dataset and excluding those with the same ICD codes from the eICU-CRD dataset.

### Outcome measures

Our primary outcome measure was patient death, defined as death in the ICU or death within 72 hours of discharge from the ICU, to account for patients who may have been discharged to hospice or transitioned to comfort measures only. There were no secondary outcomes.

## RESULTS

### Patient characteristics

In the eICU-CRD database, there were 200,859 ICU stays. After excluding 68,030 stays where the time in the ICU was less than 24 hours, 11,445 recurrent stays, 327 stays where demographic information was missing or the patient was younger than 18 years old, 59,172 elective admissions, and 65,459 ICU stays where patients died or were discharged from the ICU before Day 7, there were 7,871 ICU stays available for analysis (Figure 1).

eICU-CRD - eICU Collaborative Research Database; MIMIC-IV - Medical Information Mart for Intensive Care IV; LOS - length of stay; ICU - intensive care unit; GCS - Glasgow Coma Scale.

There were a total of 73,181 adult ICU stays in the MIMIC-IV database. After excluding 15,753 stays where the time in the ICU was less than 24 hours, 15,375 recurrent stays, 1,146 stays where GCS information was missing, 6,939 elective admissions, and 29,042 ICU stays where patients died or were discharged from the ICU before Day 7, there were 4,926 ICU stays available for analysis (Figure 1).

The cohorts of eICU-CRD patients and MIMIC-IV patients were well matched in terms of sex, age, and race, with a majority of patients being male, white, and in their early 60's (Table 1). The original cohort of patients in both databases, before the exclusion criteria were applied, were also primarily male and white (Table 4S - Supplementary Material). Patients in the eICU-CRD cohort had a lower median CCI, and fewer patients met the Sepsis 3 criteria than did those in the MIMIC-IV cohort. Patients who left the ICU before Day 7, either because of death or because they were discharged, were similar to those in the Day 7 cohort in terms of age, sex, race/ethnicity, and median CCI but were less likely to meet the Sepsis 3 criteria (Tables 5S and 6S - Supplementary Material).

**Table 1:** Demographics of the eICU Collaborative Research Database and Medical Information Mart for Intensive Care IV cohorts

		eICU-CRD n = 7,871	MIMIC-IV n = 4,926
Sex (Female)		3,396 (43.1)	2,089 (42.4)
Age		63.0 [63.0 - 73.0]	65.0 [18.0 - 98.0]
Race/ethnicity			
	Hispanic	303 (3.8)	182 (3.7)
	Black	1,064 (13.5)	427 (8.7)
	White	5,835 (74.1)	2,995 (60.8)
	Asian	137 (1.7)	121 (2.5)
	Other	532 (6.8)	1,201 (24.4)
Comorbidities			
	CCI	3.0 [3.0 - 5.0]	6.0 [6.0 - 8.0]
	Cirrhosis present	320 (4.1)	467 (9.5)
	Hypertension present	4,255 (54.1)	3,115 (63.2)
	CHF present	1,829 (23.2)	1,610 (32.7)
	Asthma present	559 (7.1)	83 (1.7)
	COPD present	1,746 (22.2)	1,151 (23.4)
	CKD stage ≥ III present	753 (9.6)	491 (10.0)
	Sepsis 3 criteria present	4,274 (54.3)	4,349 (88.3)
SOFA score			
	CNS	1.0 [1.0 - 3.0]	0.0 [0.0 - 1.0]
	Respiration	0.0 [0.0 - 2.0]	0.0 [2.0 - 3.0]
	Coagulation	0.0 [0.0 - 1.0]	0.0 [0.0 - 1.0]
	Liver	0.0 [0.0 - 0.0]	0.0 [0.0 - 0.0]
	Cardiovascular	1.0 [1.0 - 1.0]	1.0 [1.0 - 4.0]
	Renal	0.0 [0.0 - 1.0]	0.0 [0.0 - 2.0]

eICU-CRD - eICU Collaborative Research Database; MIMIC-IV - Medical Information Mart for Intensive Care IV; CCI - Charlson comorbidity index; CHF, congestive heart failure; COPD - chronic obstructive pulmonary disease; CKD, chronic kidney disease; SOFA - Sequential Organ Failure Assessment; CNS - central nervous system. After excluding stays where patients died or were discharged from the ICU before 24 hours, recurrent stays, elective admissions, patients less than 18 years old, and stays for which demographic information or GCS data were missing (see Figure 1S - Supplementary Material), the results are expressed as n (%) or median [min - max].

### Contribution of each SOFA component on Day 1 and Day 7

**Figure 2:**
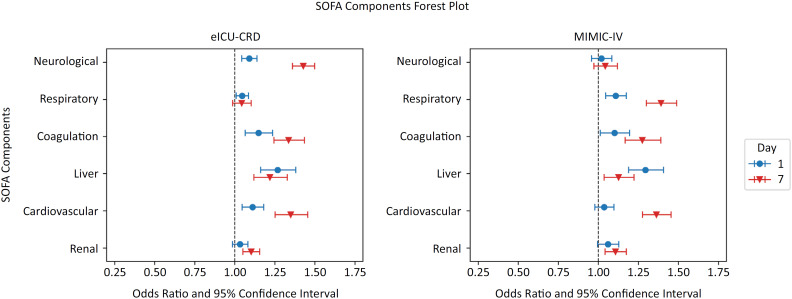
Contribution of organ dysfunction to mortality on Days 1 and 7.

In the eICU-CRD cohort, the liver component was most predictive of mortality on Day 1 (OR 1.3; 95%CI 1.2 - 1.4), followed by the CNS (OR 1.1; 95%CI 1.0 - 1.1), coagulation (OR 1.1; 95%CI 1.0 - 1.2), and cardiovascular components (OR 1.1; 95%CI 1.0 - 1.2) (Figure 2). On Day 7, the CNS component was most predictive of mortality (OR 1.4; 95%CI 1.4 - 1.5), followed by the coagulation (OR 1.3; 95%CI 1.2 - 1.4) and cardiovascular components (OR 1.3; 95%CI 1.3 - 1.5) (Table 7S - Supplementary Material). The renal and respiratory components were the least predictive of mortality on Day 1 (OR 1.0; 95%CI 1.0 - 1.1 and OR 1.0; 95%CI 1.0 - 1.1, respectively) and remained the least predictive of mortality on Day 7 (OR 1.1; 95%CI 1.1 - 1.2 and OR 1.0; 95%CI 1.0 - 1.1, respectively).

SOFA - Sequential Organ Failure Assessment; eICU-CRD - eICU Collaborative Research Database; MIMIC-IV - Medical Information Mart for Intensive Care IV.

In the MIMIC-IV cohort, the liver component was also most predictive of mortality on Day 1 (OR 1.3; 95%CI 1.2 - 1.4) (Table 8S - Supplementary Material). On Day 7, the respiratory and cardiovascular components were most predictive of mortality (OR 1.4; 95%CI 1.3 - 1.5 and OR 1.4; 95%CI 1.3 - 1.5, respectively). The CNS and cardiovascular components were least predictive of mortality on Day 1 (OR 1.0; 95%CI 1.0 - 1.1 and OR 1.0; 95%CI 1.0 - 1.1, respectively). The CNS component remained least predictive of mortality on Day 7 (OR 1.0; 95%CI 1.0 - 1.1).

In the binary variable analysis where SOFA scores were divided into freedom from organ failure (scores of 0, 1, or 2) versus organ failure (scores of 3 or 4) (Table 1S - Supplementary Material), the liver component was again the most predictive of mortality on Day 1 in both the eICU-CRD cohort (OR 2.2; 95%CI 1.6 - 2.9) and the MIMIC-IV cohort (OR 2.3; 95%CI 1.7 - 3.1) (Tables 2S and 3S - Supplementary Material). The liver component remained the most predictive of mortality on Day 7 in the eICU-CRD cohort (OR 3.0; 95%CI 2.3 - 3.8). In the MIMIC-IV cohort, respiratory dysfunction was most predictive of mortality on Day 7 (OR 3.1; 95%CI 2.5 - 3.8). The renal component was least predictive of mortality on both Day 1 and Day 7 in the eICU-CRD cohort (OR 1.2; 95%CI 1.1 - 1.4 and OR 1.5; 95%CI 1.3 - 1.8, respectively). In the MIMIC-IV cohort, CNS was least predictive of mortality on Day 1 (OR 1.0; 95%CI95% CI 0.8 - 1.3), whereas the renal component was least predictive of mortality on Day 7 (OR 1.4; 95%CI 1.1 - 1.7).

In both the discrete variable analysis and the binary variable analysis, excluding patients with cirrhosis, CHF, COPD and asthma, and CKD III (or worse) did not significantly change the contribution of the organ components to mortality (Tables 2S, 3S, 7S and 8S - Supplementary Material).

## DISCUSSION

The SOFA score has become an invaluable tool for understanding illness severity and is widely used in clinical practice and research.^(14)^ The score assigns equal weight to its six organ components when in reality, each organ's contribution to mortality may vary daily. In this study, we analyzed over 10,000 ICU stays in two different ICU datasets and found that the impact of each organ component on mortality risk was not equivalent and changed on Day 1 versus Day 7.

According to the discrete and binary variable analyses of both cohorts, liver dysfunction was most predictive of mortality on Day 1. This may be because liver function supports many other organ components, such as coagulation and renal function, and is not easily replenishable. Patients with cirrhosis can be critically ill when they arrive in the ICU. Notably, excluding patients with chronic dysfunction of one of the organ components did not change the results, suggesting that our findings reflect the impact of acute organ dysfunction and are not driven by the inclusion of patients who, at baseline, have specific organ dysfunction. Clinicians can incorporate this knowledge into their decision-making, for example, opting for treatments that minimize further liver damage. A future version of the SOFA score could incorporate the timing of organ function assessment and may more heavily weigh liver dysfunction on arrival at the ICU.

Some prior studies have shown that CNS dysfunction may be most predictive of mortality,^(7-9)^ whereas others have suggested that coagulation and cardiovascular dysfunction may be more important predictors of mortality.^(5,9,16)^ The former is consistent with our findings in the eICU-CRD cohort, whereas the latter is more consistent with our findings in the MIMIC-IV cohort, although minor differences are difficult to elicit given some overlap in our confidence intervals. These existing studies are challenging to compare given the different types of patients and ICU settings included in their analyses and different time assessments of mortality (in-ICU mortality, 30-day mortality, in-hospital mortality, etc.). The causes of organ dysfunction and mortality may differ widely across different types of specialty ICUs.^(17)^ In general, there was variability in how dysfunction of different organs contributed to mortality between our two cohorts, possibly because the two different ICU cohorts contained distinct patient populations. MIMIC-IV is a single institution dataset of patients treated at an academic tertiary care center, whereas eICU-CRD is a multi-institutional dataset of patients treated at 208 centers, including many smaller hospitals with 100 to 500 beds across the United States. Patients in the eICU-CRD dataset may also be less critically ill overall given their lower median CCI scores, smaller portion of patients who met the Sepsis 3 criteria, and shorter ICU lengths of stay.^(11)^ This finding illustrates the importance of validating the scoring system in countries with different resource and care settings, which is one of the primary principles in developing the next-generation SOFA-2 score.^(18)^

Evaluating the impact of CNS dysfunction is particularly challenging because different institutions and even different providers may score sedated and intubated patients differently. A similar study by Knox's group^(8)^ conducted a sensitivity analysis by comparing three different methods for GCS verbal scoring: an imputed verbal component in all patients, an imputed verbal component in only intubated patients, and assigning all intubated patients a verbal subscore of 1 and reported that the differing strategies did not affect their results. We similarly tried to account for variations in CNS scoring by imputing a GCS score of 15 for all intubated and sedated patients, in line with previously suggested strategies.^(14)^ However, there are limitations to the accuracy of this strategy. It is challenging to retrospectively standardize and verify the assessment of CNS dysfunction, particularly in a database that includes hundreds of different hospitals. Future studies may consider prospectively calculating a GCS score to ensure maximum accuracy and better understand how CNS dysfunction contributes to mortality.

According to the binary and discrete variable analyses of both cohorts, renal dysfunction was one of the least predictive of mortality on Days 1 and 7. This may be due to the wide availability of renal replacement therapy across ICUs. Interestingly, randomized controlled trials have shown that earlier use of renal replacement therapy and higher-intensity renal replacement therapy in critically ill patients do not reduce mortality.^(19,20)^ Given that advanced therapies have become more widely available in many ICUs, further research is needed to understand how their use can be best tailored to an individual patient. An updated SOFA score—or a completely new approach to a critical care score—that not only reflects these advances in medical care but also incorporates the day-to-day evolution of critical illness may guide clinicians in making better decisions about how and when to use potentially life-saving but high-stakes interventions.^(6,21,22)^

There are several limitations to our study. While our baseline cohorts were well matched in terms of age and sex, the breakdown of race was quite different in our Day 7 cohorts (Table 4S - Supplementary Material). This could be due to the common use of the label "other" to describe a patient's race. Future work characterizing the demographics in these datasets can help ensure that research in this area is representative of the patients we care for. We chose to include only patients who were in the ICU for at least 7 days rather than assessing patients who were alive on Day 1 versus Day 7 to minimize the risk of immortal time bias. We provided an assessment of the patients who left the ICU prior to Day 7 and showed that they were well matched to their respective Day 7 cohorts in terms of age, sex, race, ethnicity, and CCI but were less likely to meet the Sepsis 3 criteria (Tables 5S and 5S - Supplementary Material). This suggests that the final cohorts we used for analysis were more likely to be critically ill, which limits the generalizability of our results.

Our analysis was also limited in that it assessed the contribution of organ dysfunction to mortality risk on Days 1 and 7 only. We chose this strategy to test our hypothesis that the impact of organ dysfunction changes over time. However, this strategy did not allow us to account for the interplay of two organs (for example, in cardiorenal syndrome or shock liver), and the diversity of treatments patients might receive between Days 1 and 7. Interventions such as intubation, renal replacement therapy, or extracorporeal membrane oxygenation may impact how the weight of each organ component varies over time. Further work in this area should evaluate the contribution of different organ systems to mortality risk in various clinical scenarios at a higher temporal resolution.

## CONCLUSION

The SOFA score is a valuable tool for evaluating organ dysfunction and the risk of mortality in critically ill patients but may be an oversimplification in this new era of medicine. Experts are collaborating on revising the SOFA score, and further research on how the SOFA score can be improved will support their efforts in the development of the SOFA-2 score.

## Data Availability

The data that support the findings of this study are available at the MIMIC-IV (doi.org/10.1093/jamia/ocx084) and eICU-CRD (doi.org/10.1038/sdata.2018.178). Both datasets are publicly available through PhysioNet (http://physionet.org).
