# Supplementary material for: Analyzing how the components of the SOFA score change over time in their contribution to mortality
Source: Crit Care Sci. 2024 Oct 31;36:e20240030en. doi: 10.62675/2965-2774.20240030-en (PMC11634241; doi:10.62675/2965-2774.20240030-en)
Supplement: Supplementary file 1 [file 2965-2774-ccsci-36-e20240030en-Suppl01.pdf]

Critical Care Science
https://doi.org/10.62675/2965-2774.20240030-en

SUPPLEMENTARY MATERIAL

Analyzing how the components of the SOFA score change over time in their contribution to mortality

Barbara D. Lam[1], Tristan Struja[2], Yanran Li[3], João Matos[2], Ziyue Chen[4], Xiaoli Liu[2], Leo Anthony Celi[1], Yugang Jia[2], Jesse Raffa[2]

TABLE OF CONTENTS

Figure 1S - Contribution of organ dysfunction to mortality on days 1 and 7 using a binary variable analysis 2
Table 1S - SOFA organ dysfunction definitions for binary variable analysis 2
Table 2S - Contribution of organ dysfunction to mortality on day 1 and day 7; eICU-CRD binary variable analysis 3
Table 3S - Contribution of organ dysfunction to mortality on day 1 and day 7; MIMIC-IV binary variable analysis 3
Table 4S - Comparison of patient cohorts before and after exclusion criteria were applied 4
Table 5S - Comparison of day 7 cohort to patients who died or were discharged prior to day 7 4
Table 6S - Comparison of patients who died or were discharged before day 7 5
Table 7S - Contribution of organ dysfunction to mortality on day 1 and day 7; eICU-CRD discrete variable analysis 6
Table 8S - Contribution of organ dysfunction to mortality on day 1 and day 7; MIMIC-IV discrete variable analysis 6

This is an open access article under the CC BY license (https://creativecommons.org/licenses/by/4.0/).

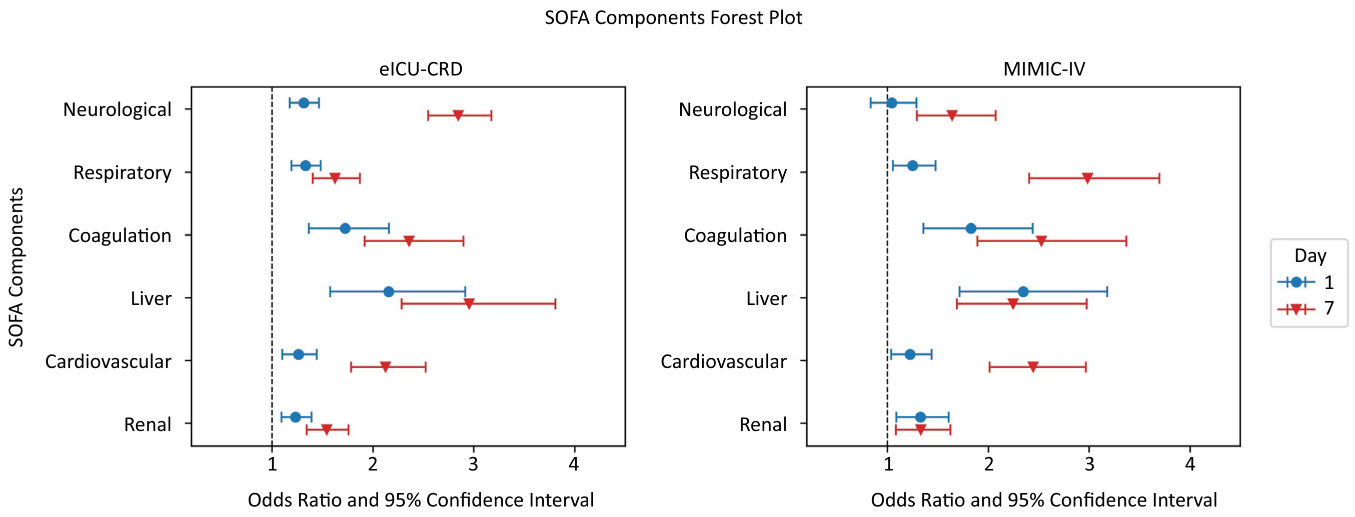

Figure 1 - Contribution of organ dysfunction to mortality on days 1 and 7 using a binary variable analysis.
SOFA - sequential organ failure assessment; eICU-CRD - eICU Collaborative Research Database; MIMIC-IV - Medical Information Mart for Intensive Care IV.

Table 1S - SOFA organ failure definitions for binary variable analysis

Organ component	Freedom from organ failure (SOFA score 0, 1, 2)	Organ failure (SOFA score 3, 4)
Respiration	$PaO_2/FiO_2 \geq$ 200mmHg	$PaO_2/FiO_2 <$ 200mmHg *or* on a ventilator
Coagulation	Platelets ≥ 50K/μL	Platelets < 50K/μL
Liver	Bilirubin < 6.0mg/dL	Bilirubin ≥ 6.0mg/dL
Cardiovascular	MAP ≥ 70mmHg<br>Dopamine ≤ 5mcg/kg/min or<br>Dobutamine at any dose	Dopamine > 5mcg/kg/min,<br>Epinephrine ≤ 0.1mcg/kg/min or<br>Norepinephrine ≤ 0.1mcg/kg/min
Central nervous system	GCS 10 - 15	GCS < 9
Renal	Creatinine < 3.5mg/dL	Creatinine ≥ 3.5mg/dL or urine output < 500mL per day

SOFA - Sequential Organ Failure Assessment; MAP - mean arterial pressure; GCS - Glasgow coma score.

Table 2S - Contribution of organ dysfunction to mortality on day 1 and day 7; eICU Collaborative Research Database binary variable analysis

SOFA score component	All patients	Excluding patients with cirrhosis	Excluding patients with CHF	Excluding patients with COPD and asthma	Excluding patients with CKD III or worse
	Odds ratio (95% confidence intervals)				
Day 1 (24 hours)					
CNS	1.3 (1.2 - 1.5)	1.3 (1.1 - 1.4)	1.3 (1.2 - 1.5)	1.3 (1.2 - 1.5)	1.3 (1.1 - 1.4)
Respiratory	1.3 (1.2 - 1.5)	1.3 (1.2 - 1.5)	1.3 (1.2 - 1.5)	1.3 (1.2 - 1.5)	1.4 (1.2 - 1.5)
Coagulation	1.7 (1.4 - 2.2)	1.8 (1.3 - 2.3)	1.8 (1.4 - 2.3)	1.8 (1.4 - 2.3)	1.9 (1.5 - 2.4)
Liver	2.2 (1.6 - 2.9)	1.7 (1.1 - 2.7)	2.3 (1.6 - 3.1)	2.0 (1.4 - 2.8)	2.1 (1.5 - 2.9)
Cardiovascular	1.3 (1.1 - 1.4)	1.3 (1.1 - 1.4)	1.4 (1.2 - 1.6)	1.3 (1.1 - 1.5)	1.3 (1.1 - 1.5)
Renal	1.2 (1.1 - 1.4)	1.2 (1.1 - 1.4)	1.3 (1.1 - 1.5)	1.2 (1.1 - 1.4)	1.3 (1.1 - 1.5)
Day 7 (168 hours)					
CNS	2.8 (2.6 - 3.2)	2.8 (2.5 - 3.1)	2.8 (2.5 - 3.1)	3.0 (2.6 - 3.4)	2.9 (2.5 - 3.3)
Respiratory	1.6 (1.4 - 1.9)	1.6 (1.4 - 1.8)	1.7 (1.4 - 1.9)	1.7 (1.5 - 2.0)	1.7 (1.4 - 2.0)
Coagulation	2.4 (1.9 - 2.9)	2.5 (2.0 - 3.1)	2.2 (1.7 - 2.7)	2.3 (1.8 - 2.9)	2.4 (1.9 - 3.0)
Liver	3.0 (2.3 - 3.8)	3.1 (2.2 - 4.2)	3.0 (2.2 - 3.9)	2.9 (2.2 - 3.8)	2.8 (2.1 - 3.8)
Cardiovascular	2.1 (1.8 - 2.5)	2.2 (1.8 - 2.6)	2.1 (1.7 - 2.5)	2.0 (1.7 - 2.5)	2.2 (1.8 - 2.7)
Renal	1.5 (1.3 - 1.8)	1.5 (1.3 - 1.7)	1.7 (1.4 - 2.0)	1.6 (1.4 - 1.8)	1.5 (1.3 - 1.8)

SOFA - Sequential Organ Failure Assessment; CHF - congestive heart failure; COPD - chronic obstructive pulmonary disease; CKD - chronic kidney disease; CNS - central nervous system.

Table 3S - Contribution of organ dysfunction to mortality on day 1 and day 7; Medical Information Mart for Intensive Care IV binary variable analysis

SOFA score component	All patients	Excluding patients with cirrhosis	Excluding patients with CHF	Excluding patients with COPD and asthma	Excluding patients with CKD III or worse
	Odds ratio (95% confidence intervals)				
Day 1 (24 hours)					
CNS	1.0 (0.8 - 1.3)	1.0 (0.8 - 1.3)	1.2 (0.9 - 1.5)	1.0 (0.8 - 1.4)	1.0 (0.8 - 1.2)
Respiratory	1.3 (1.1 - 1.5)	1.2 (1.0 - 1.5)	1.4 (1.1 - 1.7)	1.2 (1.0 - 1.5)	1.2 (1.0 - 1.4)
Coagulation	1.8 (1.4 - 2.5)	2.0 (1.4 - 2.9)	2.1 (1.5 - 3.0)	2.2 (1.6 - 3.0)	1.6 (1.2 - 2.3)
Liver	2.3 (1.7 - 3.1)	1.6 (0.9 - 2.6)	2.7 (1.9 - 3.8)	2.8 (1.9 - 3.9)	2.6 (1.9 - 3.6)
Cardiovascular	1.2 (1.0 - 1.4)	1.2 (1.0 - 1.5)	1.4 (1.1 - 1.7)	1.1 (0.9 - 1.4)	1.2 (1.0 - 1.5)
Renal	1.4 (1.1 - 1.6)	1.3 (1.1 - 1.7)	1.2 (0.9 - 1.5)	1.5 (1.2 - 1.9)	1.6 (1.3 - 2.0)
Day 7 (168 hours)					
CNS	1.6 (1.3 - 2.0)	1.6 (1.3 - 2.1)	1.5 (1.2 - 2.0)	1.7 (1.3 - 2.2)	1.5 (1.2 - 2)
Respiratory	3.1 (2.5 - 3.8)	3.0 (2.4 - 3.8)	3.4 (2.6 - 4.4)	3.3 (2.5 - 4.3)	2.9 (2.3 - 3.7)
Coagulation	2.6 (2.0 - 3.4)	2.6 (1.8 - 3.6)	2.4 (1.7 - 3.5)	2.7 (1.9 - 3.7)	2.3 (1.7 - 3.2)
Liver	2.2 (1.7 - 3.0)	2.6 (1.7 - 3.8)	2.4 (1.7 - 3.4)	2.4 (1.7 - 3.3)	2.3 (1.7 - 3.1)
Cardiovascular	2.4 (2.0 - 2.9)	2.5 (2.1 - 3.1)	2.6 (2.0 - 3.3)	2.8 (2.2 - 3.5)	2.3 (1.9 - 2.8)
Renal	1.4 (1.1 - 1.7)	1.3 (1.1 - 1.7)	1.2 (0.9 - 1.6)	1.5 (1.2 - 1.9)	1.5 (1.2 - 1.9)

MIMIC-IV - Medical Information Mart for Intensive Care IV; SOFA - Sequential Organ Failure Assessment; CHF - congestive heart failure; COPD - chronic obstructive pulmonary disease; CKD - chronic kidney disease; CNS - central nervous system.

Table 4S - Comparison of demographics before and after exclusion criteria were applied

	eICU-CRD			MIMIC-IV		
	Initial cohort (n = 200,859)	Day 1 in ICU (n = 73,330)	Day 7 in ICU (n = 10,653)	Initial cohort (n = 73,181)	Day 1 in ICU (n = 33,968)	Day 7 in ICU n = 4,926)
Sex						
Female	92,303 (46.0)	33,922 (46.3)	4,539 (42.6)	32,363 (44.2)	14,973 (44.1)	2,089 (42.4)
Race						
White	155,285 (77.3)	55,888 (76.2)	7,916 (74.3)	49,891 (68.2)	22,329 (65.7)	2,995 (60.8)
Black	21,308 (10.6)	8,826 (12.0)	1,487 (14.0)	7,960 (10.9)	3,320 (9.8)	427 (8.7)
Hispanic	7,464 (3.7)	2,678 (3.7)	354 (3.3)	2,805 (3.8)	1,240 (3.7)	182 (3.7)
Asian	3,270 (1.6)	1,215 (1.7)	210 (2.0)	2,155 (2.9)	971 (2.9)	121 (2.5)
Other	13,532 (6.7)	4,723 (6.4)	686 (6.4)	10,370 (14.2)	6,108 (18.0)	1,201 (24.4)

Before and after exclusion criteria were applied, the majority of patients in both cohorts were male and white. In both cohorts, there was a decreasing proportion of white patients by day 7. In the eICU-CRD cohort, there was an increasing proportion of black patients by day 7. In the MIMIC-IV cohort, there was an increasing proportion of patients in the “other” race group by day 7, which includes patients who identify as multiple races or other races, patients who declined to share the information, or patients where the information could not be obtained. eICU-CRD - eICU Collaborative Research Database; MIMIC-IV - Medical Information Mart for Intensive Care IV; ICU - intensive care unit. Results expressed as n (%).

Table 5S - Comparison of day 7 cohort to patients who died or were discharged prior to day 7

	eICU-CRD			MIMIC-IV		
	Day 1 in ICU (n = 73,330)	Left ICU before Day 7 (n = 65,459)	Day 7 in ICU (n = 7,871)	Day 1 in ICU (n = 33,968)	Left ICU before Day 7 (n = 29,042)	Day 7 in ICU (n = 4,926)
Sex (Female)	33,922 (46.3)	30,526 (46.6)	3,396 (43.1)	14,973 (44.1)	12,884 (44.4)	2,089 (42.4)
Age (years)	65.0	65.0	63.0	67.0	67.0	65.0
Race/ethnicity						
Hispanic	2,678 (3.7)	2,375 (3.6)	303 (3.8)	1,240 (3.7)	1,058 (3.6)	182 (3.7)
Black	8,826 (12.0)	7,762 (11.9)	1,064 (13.5)	3,320 (9.8)	2,893 (10.0)	427 (8.7)
White	55,888 (76.2)	50,053 (76.5)	5,835 (74.1)	22,329 (65.7)	19,334 (66.6)	2,995 (60.8)
Asian	1,215 (1.7)	1,078 (1.6)	137 (1.7)	971 (2.9)	850 (2.9)	121 (2.5)
Other	4,723 (6.4)	4,191 (6.4)	532 (6.8)	6,108 (18.0)	4,907 (16.9)	1,201 (24.4)
Comorbidities						
CCI	4.0 [0 - 19.0]	4.0 [4.0 - 6.0]	3.0 [3.0 - 5.0]	5.0 [0 - 20.0]	5.0 [5.0 - 8.0]	6.0 [6.0 - 8.0]
Cirrhosis present	2,629 (3.6)	2,309 (3.5)	320 (4.1)	2,341 (6.9)	1,874 (6.5)	467 (9.5)
Hypertension present	39,591 (54.0)	35,336 (54.0)	4,255 (54.1)	21,634 (63.7)	18,519 (63.8)	3,115 (63.2)
CHF present	15,212 (20.7)	13,383 (20.4)	1,829 (23.2)	9,985 (29.4)	8,375 (28.8)	1,610 (32.7)
Asthma present	5,640 (7.7)	5,081 (7.8)	559 (7.1)	395 (1.2)	312 (1.1)	83 (1.7)
COPD present	14,660 (20.0)	12,914 (19.7)	1,746 (22.2)	7,301 (21.5)	6,150 (21.2)	1,151 (23.4)
CKD stage ≥ III present	6,375 (8.7)	5,622 (8.6)	753 (9.6)	3,305 (9.7)	2,814 (9.7)	491 (10.0)
Sepsis 3 criteria present	23,980 (32.7)	19,706 (30.1)	4,274 (54.3)	17,454 (51.4)	13,105 (45.1)	4,349 (88.3)
SOFA score						
CNS	1.0 [1.0 - 3.0]	0.0 [0.0 - 1.0]	1.0 [1.0 - 3.0]	0.0 [0.0 - 1.0]	0.0 [0.0 - 1.0]	0.0 [0.0 - 1.0]
Respiration	2.0 [2.0 - 4.0]	0.0 [0.0 - 0.0]	0.0 [0.0 - 2.0]	2.0 [2.0 - 3.0]	0.0 [0.0 - 1.0]	0.0 [2.0 - 3.0]
Coagulation	0.0 [0.0 - 1.0]	0.0 [0.0 - 0.0]	0.0 [0.0 - 1.0]	0.0 [0.0 - 1.0]	0.0 [0.0 - 1.0]	0.0 [0.0 - 1.0]
Liver	0.0 [0.0 - 0.0]	0.0 [0.0 - 0.0]	0.0 [0.0 - 0.0]	0.0 [0.0 - 0.0]	0.0 [0.0 - 0.0]	0.0 [0.0 - 0.0]
Cardiovascular	0.0 [0.0 - 1.0]	1.0 [1.0 - 1.0]	1.0 [1.0 - 1.0]	1.0 [1.0 - 3.0]	1.0 [1.0 - 1.0]	1.0 [1.0 - 4.0]
Renal	0.0 [0.0 - 2.0]	0.0 [0.0 - 1.0]	0.0 [0.0 - 1.0]	0.0 [0.0 - 1.0]	0.0 [0.0 - 1.0]	0.0 [0.0 - 2.0]

eICU-CRD - eICU Collaborative Research Database; MIMIC-IV - Medical Information Mart for Intensive Care IV; ICU - intensive care unit. CC - Charlson comorbidity index; CHF - congestive heart failure; COPD - chronic obstructive pulmonary disease; CKD - chronic kidney disease; SOFA - Sequential Organ Failure Assessment; CNS - central nervous system. Results expressed as n(%), median or median [min – max].

Table 6S - Comparison of patients who died or were discharged before day 7

	eICU-CRD			MIMIC-IV		
	Death before day 7 (n = 22,008)	Discharge before day 7 (n = 43,451)	Alive in ICU at day 7 (n = 7,871)	Death before day 7 (n = 3,161)	Discharge before day 7 (n = 25,881)	Alive in ICU at day 7 (n = 4,926)
Sex (Female)	10,227 (46.5)	20,299 (46.7)	3,396 (43.1)	1,529 (48.4)	11,355 (43.9)	2,089 (42.4)
Age (years)	68.0	63.0	63.0	74.0	66.0	65.0
Race/ethnicity						
Hispanic	859 (3.9)	1,516 (3.5)	303 (3.8)	6 68 (2.2)	990 (3.8)	182 (3.7)
Black	2,701 (12.3)	5,061 (11.6)	1,064 (13.5)	241 (7.6)	2,652 (10.2)	427 (8.7)
White	16,695 (75.9)	33,358 (76.8)	5,835 (74.1)	1,978 (62.6)	17,356 (67.1)	2,995 (60.8)
Asian	397 (1.8)	681 (1.6)	137 (1.7)	104 (3.3)	746 (2.9)	121 (2.5)
Other	1,356 (6.2)	2,835 (6.5)	532 (6.8)	770 (24.4)	4,137 (16.0)	1,201 (24.4)
Comorbidities						
CCI	4.0 [4.0 - 6.0]	3.0 [3.0 - 5.0]	3.0 [3.0 - 5.0]	7.0 [7.0 - 9.0]	5.0 [5.0 - 7.0]	6.0 [6.0 - 8.0]
Cirrhosis present	938 (4.3)	1,371 (3.2)	324 (4.1)	385 (12.2)	1,489 (5.8)	467 (9.5)
Hypertension present	12,345 (56.1)	22,991 (52.9)	4,255 (54.1)	2,055 (65.0)	16,464 (63.6)	3,115 (63.2)
CHF present	5,417 (24.6)	7,966 (18.3)	1,829 (23.2)	1,120 (35.4)	7,255 (28.0)	1,610 (32.7)
Asthma present	1,548 (7.0)	3,533 (81)	559 (7.1)	70 (2.2)	242 (0.9)	83 (1.7)
COPD present	4,768 (21.7)	8,146 (18.7)	1,746 (22.2)	742 (23.5)	5,408 (20.9)	1,151 (23.4)
CKD stage ≥ III present	2,394 (10.9)	3,228 (7.4)	753 (9.6)	392 (12.4)	2,422 (9.4)	491 (10.0)
Sepsis 3 criteria present	9,415 (42.8)	10,291 (23.7)	4,274 (54.3)	2,095 (66.3)	11,010 (42.5)	4,349 (88.3)
SOFA score						
CNS	0.0 [0.0 - 2.0]	0.0 [0.0 - 1.0]	2.0 [2.0 - 3.0]	0.0 [0.0 - 2.0]	0.0 [0.0 - 1.0]	0.0 [0.0 - 1.0]
Respiration	0.0 [0.0 - 2.0]	0.0 [0.0 - 0.0]	2.0 [2.0 - 4.0]	0.0 [0.0 - 2.0]	2.0 [0.0 - 1.0]	0.0 [2.0 - 3.0]
Coagulation	0.0 [0.0 - 1.0]	0.0 [0.0 - 0.0]	0.0 [0.0 - 1.0]	0.0 [0.0 - 1.0]	0.0 [0.0 - 1.0]	0.0 [0.0 - 1.0]
Liver	0.0 [0.0 - 0.0]	0.0 [0.0 - 0.0]	0.0 [0.0 - 0.0]	0.0 [0.0 - 1.0]	0.0 [0.0 - 0.0]	0.0 [0.0 - 0.0]
Cardiovascular	1.0 [1.0 - 1.0]	1.0 [1.0 - 1.0]	1.0 [1.0 - 1.0]	1.0 [1.0 - 4.0]	1.0 [1.0 - 1.0]	1.0 [1.0 - 4.0]
Renal	1.0 [1.0 - 2.0]	1.0 [1.0 - 2.0]	1.0 [1.0 - 1.0]	1.0 [1.0 - 2.0]	0.0 [0.0 - 1.0]	0.0 [0.0 - 2.0]

eICU-CRD - eICU Collaborative Research Database; MIMIC-IV - Medical Information Mart for Intensive Care IV; ICU - intensive care unit. CCI - Charlson comorbidity index; CHF - congestive heart failure; COPD - chronic obstructive pulmonary disease; CKD - chronic kidney disease; SOFA - Sequential Organ Failure Assessment; CNS - central nervous system. Results expressed as n (%), median or median [min – max].

Table 7S - Contribution of organ dysfunction to mortality on day 1 and day 7; eICU Collaborative Research Database discrete variable analysis

SOFA score component	All patients (n = 7,871)	Excluding patients with cirrhosis (n = 7,551)	Excluding patients with CHF (n = 6,042)	Excluding patients with COPD and asthma (n = 5,883)	Excluding patients with CKD III or worse (n = 7118)
	Odds Ratio (95% confidence intervals)				
Day 1 (24 hours)					
CNS	1.1 (1.0 - 1.1)	1.1 (1.0 - 1.1)	1.1 (1.01.2)	1.1 (1.1 - 1.2)	1.1 (1.0 - 1.1)
Respiratory	1.0 (1.0 - 1.1)	1.0 (1.0 - 1.1)	1.0 (1.0 - 1.1)	1.0 (1.0 - 1.1)	1.0 (1.0 - 1.1)
Coagulation	1.1 (1.0 - 1.2)	1.1 (1.0 - 1.2)	1.1 (1.0 - 1.2)	1.1 (1.0 - 1.2)	1.1 (1.1 - 1.2)
Liver	1.3 (1.2 - 1.4)	1.2 (1.1 - 1.3)	1.3 (1.2 - 1.4)	1.2 (1.1 - 1.3)	1.3 (1.1 - 1.4)
Cardiovascular	1.1 (1.0 - 1.2)	1.1 (1.0 - 1.2)	1.2 (1.1 - 1.2)	1.1 (1.0 - 1.2)	1.1 (1.1 - 1.2)
Renal	1.0 (1.0 - 1.1)	1.0 (1.0 - 1.1)	1.0 (1.0 - 1.1)	1.0 (1.0 - 1.1)	1.0 (1.0 - 1.1)
Day 7 (168 hours)					
CNS	1.4 (1.4 - 1.5)	1.4 (1.4 - 1.5)	1.4 (1.3 - 1.5)	1.5 (1.4 - 1.6)	1.4 (1.3 - 1.5)
Respiratory	1.0 (1.0 - 1.1)	1.0 (1.0 - 1.1)	1.1 (1.0 - 1.1)	1.1 (1.0 - 1.1)	1.0 (1.0 - 1.1)
Coagulation	1.3 (1.2 - 1.4)	1.3 (1.2 - 1.4)	1.4 (1.3 - 1.5)	1.3 (1.2 - 1.4)	1.3 (1.2 - 1.4)
Liver	1.2 (1.1 - 1.3)	1.2 (1.1 - 1.4)	1.2 (1.1 - 1.3)	1.2 (1.1 - 1.3)	1.2 (1.1 - 1.3)
Cardiovascular	1.3 (1.3 - 1.5)	1.3 (1.2 - 1.4)	1.4 (1.3 - 1.5)	1.4 (1.3 - 1.5)	1.3 (1.2 - 1.5)
Renal	1.1 (1.1 - 1.2)	1.1 (1.0 - 1.2)	1.1 (1.0 - 1.2)	1.1 (1.1 - 1.2)	1.1 (1.1 - 1.2)

SOFA - Sequential Organ Failure Assessment; CHF - congestive heart failure; COPD - chronic obstructive pulmonary disease; CKD, chronic kidney disease; CNS - central nervous system.

Table 8S - Contribution of organ dysfunction to mortality on day 1 and day 7; Medical Information Mart for Intensive Care IV discrete variable analysis

SOFA score component	All patients (n = 4,926)	Excluding patients with cirrhosis (n = 4,459)	Excluding patients with CHF (n = 3,316)	Excluding patients with COPD and asthma (n = 3,726)	Excluding patients with CKD III or worse (n = 4,435)
	Odds Ratio (95% confidence intervals)				
Day 1 (24 hours)					
CNS	1.0 (1.0 - 1.1)	1.0 (1.0 - 1.1)	1.1 (1.0 - 1.1)	1.0 (1.0 -1 .1)	1.0 (1.0 - 1.1)
Respiratory	1.1 (1.0 - 1.2)	1.1 (1.0 - 1.2)	1.1 (1.0 - 1.2)	1.1 (1.0 - 1.2)	1.1 (1.0 - 1.2)
Coagulation	1.1 (1.0 - 1.2)	1.1 (1.0 - 1.2)	1.1 (1.0 - 1.3)	1.2 (1.1 - 1.3)	1.1 (1.0 - 1.2)
Liver	1.3 (1.2 - 1.4)	1.2 (1.1 - 1.3)	1.3 (1.2 - 1.5)	1.3 (1.2 - 1.5)	1.3 (1.2 - 1.4)
Cardiovascular	1.0 (1.0 - 1.1)	1.0 (1.0 - 1.1)	1.1 (1.0 - 1.2)	1.0 (1.0 - 1.1)	1.0 (1.0 - 1.1)
Renal	1.1 (1.0 - 1.1)	1.1 (1.0 - 1.1)	1.0 (1.0 - 1.1)	1.1 (1.0 - 1.2)	1.1 (1.0 - 1.2)
Day 7 (168 hours)					
CNS	1.0 (1.0 - 1.1)	1.0 (1.0 - 1.1)	1.0 (1.0 - 1.1)	1.0 (1.0 - 1.1)	1.0 (1.0 - 1.1)
Respiratory	1.4 (1.3 - 1.5)	1.4 (1.3 - 1.5)	1.4 (1.3 - 1.5)	1.4 (1.2 - 1.5)	1.4 (1.3 - 1.5)
Coagulation	1.3 (1.2 - 1.4)	1.2 (1.1 - 1.4)	1.3 (1.1 - 1.4)	1.3 (1.2 - 1.4)	1.3 (1.1 - 1.4)
Liver	1.1 (1.0 - 1.2)	1.1 (1.0 - 1.2)	1.1 (1.0 - 1.3)	1.1 (1.0 - 1.3)	1.1 (1.0 - 1.2)
Cardiovascular	1.4 (1.3 - 1.5)	1.4 (1.3 - 1.5)	1.4 (1.3 - 1.5)	1.4 (1.3 - 1.5)	1.4 (1.3 - 1.4)
Renal	1.1 (1.0 - 1.2)	1.1 (1.0 - 1.2)	1.1 (1.0 - 1.2)	1.1 (1.1 - 1.2)	1.1 (1.1 - 1.2)

SOFA - Sequential Organ Failure Assessment; CHF - congestive heart failure; COPD - chronic obstructive pulmonary disease; CKD, chronic kidney disease; CNS - central nervous system.
