# Supplementary material for: Analyzing how the components of the SOFA score change over time in their contribution to mortality
Source: Crit Care Sci. 2024 Oct 31;36:e20240030en. doi: 10.62675/2965-2774.20240030-en (PMC11634241; doi:10.62675/2965-2774.20240030-en)
Supplement: Translation

# Translation: Análise de como os componentes do escore SOFA variam ao longo do tempo em sua contribuição para a mortalidade

**DOI:** 10.62675/2965-2774.20240030-pt

**Authors:** Barbara D. Lam, Tristan Struja, Yanran Li, João Matos, Ziyue Chen, Xiaoli Liu, Leo Anthony Celi, Yugang Jia, Jesse Raffa

**Affiliations:** 1 Boston MA United States Department of Medicine, Beth Israel Deaconess Medical Center - Boston (MA), United States.; 2 Cambridge MA United States Laboratory for Computational Physiology, Institute for Medical Engineering and Science, Massachusetts Institute of Technology - Cambridge (MA), United States.; 3 Boston MA United States Harvard T.H. Chan School of Public Health - Boston (MA), United States.; 4 Singapore Genome Institute of Singapore, Agency for Science, Technology and Research – Singapore.

**Keywords:** Escores de disfunção orgânica, Alta do paciente, Modelos logísticos, Fígado, Sistema nervoso central, Mortalidade, Hospitais, Unidades de terapia intensiva

## Abstract

**Objetivo:**

Determinar como cada componente de órgão do escore SOFA difere em sua contribuição no risco de mortalidade e como essa contribuição pode variar ao longo do tempo.

**Métodos:**

Realizamos uma análise de regressão logística multivariada para avaliar a contribuição de cada componente de órgão no risco de mortalidade no primeiro e sétimo dia de internação em unidade de terapia intensiva. Usamos dados de dois conjuntos de dados públicos, a *eICU* *Collaborative Research Database* (eICU-CRD) (208 hospitais) e a *Medical Information Mart for Intensive Care IV* (MIMIC-IV) (um hospital). Calculamos a razão de chances de cada componente do SOFA, que contribuiu para a mortalidade. A mortalidade foi definida como morte na unidade de terapia intensiva ou dentro de 72 horas após a alta da unidade de terapia intensiva.

**Resultados:**

Foram incluídas 7.871 internações em unidades de terapia intensiva da eICU-CRD e 4.926 internações em unidades de terapia intensiva da MIMIC-IV. A disfunção hepática foi a mais preditiva de mortalidade no primeiro dia em ambas as coortes (RC 1,3; IC95% 1,2 - 1,4; RC 1,3; IC95% 1,2 - 1,4, respectivamente). Na coorte eICU-CRD, a disfunção do sistema nervoso central foi a mais preditiva de mortalidade no sétimo dia (RC 1,4; IC95% 1,4 - 1,5). Na coorte MIMIC-IV, a disfunção respiratória (RC 1,4; IC95% 1,3 - 1,5) e cardiovascular (RC 1,4; IC95% 1,3 - 1,5) foram mais preditivas de mortalidade no sétimo dia.

**Conclusão:**

O escore SOFA pode ser uma simplificação exagerada de como a disfunção de diferentes sistemas de órgãos contribui para a mortalidade ao longo do tempo. São necessários mais estudos em uma escala de tempo mais granular para explorar como o escore SOFA pode evoluir e ser aprimorado.

## INTRODUÇÃO

A importância do escore *Sequential Organ Failure Assessment* (SOFA) foi enfatizada por sua inclusão nas diretrizes Sepsis 3 de 2016 como parte dos critérios para identificar pacientes com sepse.^(1)^ Um escore SOFA mais alto, o escore SOFA máximo e o padrão de sua variação demonstraram ser preditores confiáveis de mortalidade.^(2-5)^ Houve avanços significativos na medicina de cuidados intensivos desde que o escore SOFA foi instituído, há mais de 25 anos; e agora os especialistas pedem que o escore seja reavaliado e modernizado.^(6)^

Historicamente, os seis componentes de órgãos do escore SOFA - respiratório, hematológico, renal, hepático, neurológico e cardiovascular - eram igualmente ponderados. Mais recentemente, pesquisadores levantaram a hipótese de que sistemas de órgãos específicos podem desempenhar papel mais importante do que outros, com alguns achados sugerindo que a pontuação da escala de coma de Glasgow, um reflexo da disfunção neurológica, é o mais importante preditor de mortalidade.^(7-10)^ Esses estudos anteriores usaram coortes de um único centro e avaliaram como a disfunção de cada componente de órgão em um dado momento contribuiu para a mortalidade.

Neste estudo, nosso objetivo foi expandir o trabalho anterior determinando como cada componente de órgão do escore SOFA difere em sua contribuição no risco de mortalidade e como essa contribuição pode variar ao longo do tempo

## MÉTODOS

### Desenho do estudo

Calculamos o escore SOFA de cada componente de órgão em 24 horas (denominado primeiro dia) e 168 horas (denominado sétimo dia) e avaliamos como o escore de cada componente de órgão contribuiu para a mortalidade. A análise foi realizada primeiramente em uma coorte de pacientes de um centro de assistência terciária e, em seguida, replicada em uma coorte de pacientes de um banco de dados multicêntrico para comparação.

### Fonte de dados

Usamos dois conjuntos de dados de unidade de terapia intensiva (UTI) para realizar nosso estudo de coorte retrospectivo: o banco de dados *Medical Information Mart for Intensive Care IV* (MIMIC-IV)^(11)^ e a *eICU Collaborative Research Database* (eICU-CRD).^(12)^ O MIMIC-IV é um banco de dados público que contém informações de pacientes reais internados em UTIs em um centro médico acadêmico terciário, o *Beth Israel Deaconess Medical Center* (BIDMC), em Boston, Estados Unidos, entre 2008 e 2019. Os dados do MIMIC-IV foram previamente desidentificados, e os conselhos de revisão institucional do *Massachusetts Institute of Technology* (nº 0403000206) e do BIDMC (2001-P-001699/14) aprovaram o uso do banco de dados para a pesquisa. O eICU-CRD é um banco de dados multicêntrico público, proveniente do Programa de Telessaúde de eICU da Philips Healthcare e contém informações de mais de 200 mil admissões em UTIs monitoradas por programas de eICU-CRD nos Estados Unidos. Os dados da eICU-CRD não abrangem os dados da MIMIC-IV. O uso do banco de dados eICU-CRD está isento de aprovação do conselho de revisão institucional devido ao projeto retrospectivo, à falta de intervenção direta do paciente e ao esquema de segurança, para o qual o risco de reidentificação foi certificado como atendendo aos padrões de porto seguro por um especialista em privacidade independente (Privacert, Cambridge, MA; Health Insurance Portability and Accountability Act Certification No. 1031219-2). Ambos os bancos de dados contêm informações abrangentes das internações na UTI, incluindo sinais vitais, exames laboratoriais, medicamentos e dados de mortalidade.

### Definições de disfunção orgânica

O escore SOFA consiste em seis componentes de órgãos: sistema nervoso central (SNC), respiratório, hematológico, hepático, renal e cardiovascular. Cada componente é pontuado em uma escala de zero a quatro, em que zero indica nenhuma disfunção orgânica e as pontuações de um a quatro indicam piora da disfunção orgânica.

Usamos a contagem de plaquetas para definir as anormalidades de coagulação, o nível de bilirrubina para definir as anormalidades hepáticas, o nível de creatinina para definir as anormalidades renais, a pressão arterial média (PAM) e/ou a dose de vasopressores para definir as anormalidades cardiovasculares e a relação entre pressão parcial de oxigênio e fração inspirada de oxigênio (PaO_2_/FiO_2_) para definir as anormalidades respiratórias. A escala de coma de Glasgow é usada no componente SNC. Considerando que nosso conjunto de dados inclui centenas de hospitais e que a escala de coma de Glasgow é conhecida por ser medida de forma heterogênea e imprecisa, especialmente em diferentes instituições,^(13)^ imputamos uma pontuação de escala de coma de Glasgow de 15 para todos os pacientes que foram intubados ou sedados, conforme estratégias anteriores delineadas nesse campo de pesquisa.^(14,15)^

### Análise estatística

Realizamos uma análise de regressão logística multivariada para avaliar a contribuição de cada componente de órgão na mortalidade no primeiro e no sétimo dia. Ajustamos os possíveis fatores de confusão, incluindo idade, sexo, cor/etnia e estado de saúde inicial, por meio do índice de comorbidade de Charlson (ICC). Calcularam-se as razões de chances (RCs) com intervalos de confiança de 95% (IC95%). Em seguida, realizamos uma análise de sensibilidade para avaliar como a presença de pacientes com doenças crônicas comuns envolvendo diferentes componentes de órgãos pode afetar os resultados. Removemos pacientes com insuficiência cardíaca congestiva, cirrose, doença pulmonar obstrutiva crônica (DPOC), asma e doença renal crônica (DRC) estágio III ou pior e reanalisamos as contribuições de cada componente de órgão relevante para a mortalidade.

Estudos anteriores nesse campo de pesquisa usaram um sistema de pontuação binária em que um escore SOFA de zero, um ou dois representa ausência de falência de órgãos, enquanto um escore três ou quatro representa falência de órgãos.^(8,9,16)^ Inicialmente, realizamos uma análise com base nesse sistema de pontuação binária, e os resultados detalhados são apresentados na figura 1S e nas tabelas 1S - 3S (Material Suplementar). No entanto, para incluir melhor os efeitos da disfunção orgânica leve, realizamos outra análise usando variáveis SOFA discretas (0, 1, 2, 3, 4) e também apresentamos esses achados a seguir.

### Seleção da coorte

Incluímos pacientes com 18 anos ou mais internados na UTI por pelo menos 168 horas (7 dias) e com dados demográficos e escore SOFA disponíveis. Se os pacientes tiverem sido admitidos na UTI várias vezes, incluímos apenas a primeira internação que atendeu aos critérios de inclusão. As internações eletivas foram excluídas por meio da identificação dos códigos da Classificação Internacional de Doenças (CID) associados a internação eletiva no conjunto de dados MIMIC-IV e da exclusão daqueles com os mesmos códigos CID do conjunto de dados eICU-CRD.

### Medidas de desfechos

Nosso desfecho primário foi a morte do paciente, definida como morte na UTI ou morte dentro de 72 horas após a alta da UTI, para contabilizar os pacientes que podem ter recebido alta para uma clínica paliativa ou que passaram apenas para medidas de conforto. Não houve desfechos secundários.

## RESULTADOS

### Características dos pacientes

No banco de dados eICU-CRD, foram registradas 200.859 internações na UTI. Após a exclusão de 68.030 internações em que a internação na UTI foi inferior a 24 horas; 11.445 internações recorrentes; 327 internações em que as informações demográficas estavam ausentes ou o paciente tinha menos de 18 anos; 59.172 internações eletivas; e 65.459 internações na UTI em que os pacientes morreram ou receberam alta da UTI antes do sétimo dia, restaram 7.871 internações na UTI para análise (Figura 1).

**Figura 1:** Seleção de coorte dos bancos de dados da *eICU Collaborative Research Database* e do *Medical Information Mart for Intensive Care IV*.

UTI - unidade de terapia intensiva; eICU-CRD - *eICU Collaborative Research Database*; MIMIC-IV - *Medical Information Mart for Intensive Care IV*; ECG - escala de coma de Glasgow.

O banco de dados MIMIC-IV contém 73.181 internações em UTIs adulto. Após a exclusão de 15.753 internações em que a duração foi inferior a 24 horas, 15.375 internações recorrentes, 1.146 internações em que faltavam informações sobre a escala de coma de Glasgow, 6.939 internações eletivas e 29.042 internações na UTI em que os pacientes morreram ou receberam alta da UTI antes do sétimo dia, restaram 4.926 internações na UTI para análise (Figura 1).

As coortes de pacientes do eICU-CRD e de pacientes do MIMIC-IV foram bem combinadas em termos de sexo, idade e cor/etnia, com a maioria dos pacientes sendo do sexo masculino, brancos e com 60 anos ou mais (Tabela 1). A coorte original de pacientes em ambos os bancos de dados, antes da aplicação dos critérios de exclusão, também era majoritariamente masculina e branca (Tabela 4S - Material suplementar). Os pacientes da coorte eICU-CRD tinham mediana de ICC mais baixa e menos pacientes atendiam aos critérios Sepsis 3 do que os da coorte MIMIC-IV. Os pacientes que deixaram a UTI antes do sétimo dia, seja por morte ou por terem recebido alta, eram semelhantes aos da coorte do sétimo dia em termos de idade, sexo, cor/etnia e mediana de ICC, mas tinham menor probabilidade de atender aos critérios Sepsis 3 (Tabelas 5S e 6S - Material suplementar).

**Tabela 1:** Dados demográficos das coortes da *eICU Collaborative Research Database* e do *Medical Information Mart for Intensive Care IV*

		eICU-CRD n = 7.871	MIMIC-IV n = 4.926
Sexo feminino		3.396 (43,1)	2.089 (42,4)
Idade		63,0 [63,0 - 73,0]	65,0 [18,0 - 98,0]
Cor/etnia			
	Hispânica	303 (3,8)	182 (3,7)
	Negra	1.064 (13,5)	427 (8,7)
	Branca	5.835 (74,1)	2.995 (60,8)
	Asiática	137 (1,7)	121 (2,5)
	Outras	532 (6,8)	1.201 (24,4)
Comorbidades			
	ICC	3,0 [3,0 - 5,0]	6,0 [6,0 - 8,0]
	Cirrose	320 (4,1)	467 (9,5)
	Hipertensão	4.255 (54,1)	3.115 (63,2)
	Insuficiência cardíaca congestiva	1.829 (23,2)	1.610 (32,7)
	Asma	559 (7,1)	83 (1,7)
	DPOC	1.746 (22,2)	1.151 (23,4)
	Estágio de DRC ≥ III	753 (9,6)	491 (10,0)
	Critérios Sepsis 3	4.274 (54,3)	4.349 (88,3)
Escore SOFA			
	SNC	1,0 [1,0 - 3,0]	0,0 [0,0 - 1,0]
	Respiratório	0,0 [0,0 - 2,0]	0,0 [2,0 - 3,0]
	Hematológico	0,0 [0,0 - 1,0]	0,0 [0,0 - 1,0]
	Hepático	0,0 [0,0 - 0,0]	0,0 [0,0 - 0,0]
	Cardiovascular	1,0 [1,0 - 1,0]	1,0 [1,0 - 4,0]
	Renal	0,0 [0,0 - 1,0]	0,0 [0,0 - 2,0]

eICU-CRD - *eICU Collaborative Research Database*; MIMIC-IV - *Medical Information Mart for Intensive Care IV*; ICC – índice de comorbidade de Charlson; DPOC - doença pulmonar obstrutiva crônica; DRC - doença renal crônica; SOFA - *Sequential Organ Failure Assessment*; SNC - sistema nervoso central. Depois de excluir as internações em que os pacientes morreram ou receberam alta da unidade de terapia intensiva antes de 24 horas, internações recorrentes, internações eletivas, pacientes com menos de 18 anos e internações para as quais faltavam informações demográficas ou dados da escala de coma de Glasgow (vide Figura 1S - Material Suplementar), os resultados são expressos como n (%) ou mediana [25% - 75%].

### Contribuição de cada componente do SOFA no primeiro e no sétimo dia

Na coorte eICU-CRD, o componente hepático foi o mais preditivo de mortalidade no primeiro dia (RC de 1,3; IC95% 1,2 - 1,4), seguido pelo SNC (RC de 1,1; IC95% 1,0 - 1,1), hematológico (RC de 1,1; IC95% 1,0 - 1,2) e componentes cardiovasculares (RC de 1,1; IC95% 1,0 - 1,2) (Figura 2). No sétimo dia, o componente SNC foi o mais preditivo de mortalidade (RC de 1,4; IC95% 1,4 - 1,5), seguido pelos componentes hematológico (RC de 1,3; IC95% 1,2 - 1,4) e cardiovascular (RC de 1,3; IC95% 1,3 - 1,5) (Tabela 7S - Material suplementar). Os componentes renal e respiratório foram os menos preditivos de mortalidade no primeiro dia (RC de 1,0; IC95% 1,0 - 1,1 e RC de 1,0; IC95% 1,0 - 1,1, respectivamente) e continuaram sendo os menos preditivos de mortalidade no sétimo dia (RC de 1,1; IC95% 1,1 - 1,2 e RC de 1,0; IC95% 1,0 - 1,1, respectivamente).

**Figura 2:** Contribuição da disfunção orgânica na mortalidade no primeiro e no sétimo dia.

SOFA - *Sequential Organ Failure Assessment*; eICU-CRD - *eICU Collaborative Research Database*; MIMIC-IV - *Medical Information Mart for Intensive Care IV*.

Na coorte MIMIC-IV, o componente hepático também foi o mais preditivo de mortalidade no primeiro dia (RC de 1,3; IC95% 1,2 - 1,4) (Tabela 8S - Material suplementar). No sétimo dia, os componentes respiratório e cardiovascular foram os mais preditivos de mortalidade (RC de 1,4; IC95% 1,3 - 1,5 e RC de 1,4; IC95% 1,3 - 1,5, respectivamente). Os componentes SNC e cardiovascular foram os menos preditivos de mortalidade no primeiro dia (RC de 1,0; IC95% 1,0 - 1,1 e RC de 1,0; IC95% 1,0 - 1,1, respectivamente). O componente SNC continuou sendo o menos preditivo de mortalidade no sétimo dia (RC de 1,0; IC95% 1,0 - 1,1, respectivamente).

Na análise de variáveis binárias, em que os escores SOFA foram divididos em ausência de falência de órgãos (escores de zero, um ou dois) *versus* falência de órgãos (escores três ou quatro) (Tabela 1S - Material Suplementar), o componente hepático foi novamente o fator mais preditivo de mortalidade no primeiro dia, na coorte eICU-CRD (RC de 2,2; IC95% 1,6 - 2,9) e na coorte MIMIC-IV (RC de 2,3; IC95% 1,7 - 3,1) (Tabelas 2S e 3S - Material suplementar). O componente hepático continuou sendo o mais preditivo de mortalidade no sétimo dia na coorte eICU-CRD (RC de 3,0; IC95% 2,3 - 3,8). Na coorte MIMIC-IV, a disfunção respiratória foi o fator mais preditivo de mortalidade no sétimo dia (RC de 3,1; IC95% 2,5 - 3,8). O componente renal foi o menos preditivo de mortalidade tanto no primeiro quanto no sétimo dia na coorte eICU-CRD (RC de 1,2; IC95% 1,1 - 1,4 e RC de 1,5; IC95% 1,3 - 1,8, respectivamente). Na coorte MIMIC-IV, o SNC foi o menos preditivo de mortalidade no primeiro dia (RC de 1,0; IC95% 0,8 - 1,3), enquanto o componente renal foi o menos preditivo de mortalidade no sétimo dia (RC de 1,4; IC95% 1,1 - 1,7).

Tanto na análise de variável discreta quanto na análise de variável binária, a exclusão de pacientes com cirrose, insuficiência cardíaca congestiva, DPOC e asma e DRC III (ou pior) não alterou significativamente a contribuição dos componentes do órgão na mortalidade (Tabelas 2S, 3S, 7S e 8S - Material Suplementar).

## DISCUSSÃO

O escore SOFA tornou-se uma ferramenta valiosa para compreender a gravidade da doença e é amplamente utilizado na prática clínica e em pesquisas.^(14)^ O escore atribui peso igual aos seus seis componentes de órgãos quando, na realidade, a contribuição de cada órgão na mortalidade pode variar diariamente. Neste estudo, analisamos mais de 10 mil internações em UTIs em dois conjuntos de dados de UTIs diferentes e descobrimos que o impacto de cada componente de órgão no risco de mortalidade não era equivalente e variava no primeiro e no sétimo dia.

De acordo com as análises de variáveis discretas e binárias de ambas as coortes, a disfunção hepática foi a mais preditiva de mortalidade no primeiro dia. Isso pode ser devido ao fato de a função hepática apoiar muitos outros componentes do órgão, como a coagulação e a função renal, e não ser facilmente reabastecida. Os pacientes com cirrose podem estar em estado crítico quando chegam à UTI. Notavelmente, a exclusão de pacientes com disfunção crônica de um dos componentes do órgão não alterou os resultados, o que sugere que nossos achados refletem o impacto da disfunção aguda do órgão e não são impulsionados pela inclusão de pacientes que, a princípio, têm disfunção específica do órgão. Os clínicos podem incorporar esse conhecimento em suas tomadas de decisão, por exemplo, optando por tratamentos que minimizem outros danos hepáticos. Uma versão futura do escore SOFA poderia incorporar o momento da avaliação da função dos órgãos e pode ponderar mais a disfunção hepática na chegada à UTI.

Alguns estudos anteriores mostraram que a disfunção do SNC pode ser o maior preditor de mortalidade,^(7-9)^ enquanto outros sugeriram que a coagulação e a disfunção cardiovascular podem ser preditores mais importantes de mortalidade.^(5,9,16)^ O primeiro é consistente com nossos achados na coorte eICU-CRD, enquanto o último é mais consistente com nossos achados na coorte MIMIC-IV, embora seja difícil identificar pequenas diferenças, dada a sobreposição de nossos intervalos de confiança. Esses estudos existentes são difíceis de comparar devido aos diferentes tipos de pacientes e ambientes de UTI incluídos em suas análises e diferentes avaliações de tempo de mortalidade (mortalidade na UTI, mortalidade em 30 dias, mortalidade hospitalar etc.). As causas de disfunção orgânica e mortalidade podem diferir amplamente entre os diferentes tipos de UTIs especializadas.^(17)^ Em geral, houve variabilidade na forma como a disfunção de diferentes órgãos contribuiu para a mortalidade entre nossas duas coortes, possivelmente porque as duas coortes de UTIs diferentes continham populações de pacientes distintas. O MIMIC-IV é um conjunto de dados de uma única instituição de pacientes tratados em um centro acadêmico de assistência terciária, enquanto o eICU-CRD é um conjunto de dados multi-institucional de pacientes tratados em 208 centros, incluindo muitos hospitais com 100 a 500 leitos nos Estados Unidos. Os pacientes do conjunto de dados da eICU-CRD também podem estar em estado menos crítico em geral, devido às suas pontuações medianas mais baixas de ICC, à menor parcela de pacientes que atenderam aos critérios Sepsis 3 e ao menor tempo de internação na UTI.^(11)^ Esse achado ilustra a importância de validar o sistema de pontuação em países com diferentes recursos e configurações de assistência, o que é um dos princípios primários no desenvolvimento da próxima geração da pontuação SOFA-2.^(18)^

A avaliação do impacto da disfunção do SNC é particularmente desafiadora porque diferentes instituições e até mesmo diferentes provedores podem pontuar pacientes sedados e intubados de forma diferente. Um estudo semelhante realizado pelo grupo Knox^(8)^ realizou uma análise de sensibilidade comparando três métodos diferentes da pontuação verbal da escala de coma de Glasgow: um componente verbal imputado em todos os pacientes, um componente verbal imputado apenas em pacientes intubados e a atribuição de uma subpontuação verbal de 1 a todos os pacientes intubados, e relatou que as diferentes estratégias não afetaram seus resultados. Da mesma forma, tentamos considerar as variações na pontuação do SNC imputando uma pontuação de 15 na escala de coma de Glasgow para todos os pacientes intubados e sedados, segundo as estratégias sugeridas anteriormente.^(14)^ No entanto, há limitações quanto à precisão dessa estratégia. É um desafio padronizar e verificar retrospectivamente a avaliação da disfunção do SNC, especialmente em um banco de dados que inclui centenas de hospitais diferentes. Estudos futuros podem considerar o cálculo prospectivo de uma pontuação da escala de coma de Glasgow para garantir a máxima precisão e entender melhor como a disfunção do SNC contribui para a mortalidade.

De acordo com as análises de variáveis binárias e discretas de ambas as coortes, a disfunção renal foi uma das menos preditivas de mortalidade no primeiro e no sétimo dia. Isso provavelmente se deve à ampla disponibilidade de terapia de substituição renal nas UTIs. Curiosamente, estudos controlados e randomizados mostraram que o uso precoce da terapia de substituição renal e a terapia de substituição renal de maior intensidade em pacientes graves não reduzem a mortalidade.^(19,20)^ Como as terapias avançadas se tornaram mais amplamente disponíveis em muitas UTIs, são necessárias mais pesquisas para entender como seu uso pode ser melhor adaptado a um determinado paciente. Um escore SOFA atualizado—ou uma abordagem completamente nova para um escore de cuidados intensivos—que não apenas reflita esses avanços na assistência médica, mas também incorpore a evolução diária da doença crítica, pode orientar os clínicos a tomar melhores decisões sobre como e quando usar intervenções com potencial de salvar vidas, mas de alto risco.^(6,21,22)^

Nosso estudo apresenta várias limitações. Embora nossas coortes basais tenham sido bem combinadas em termos de idade e sexo, a discriminação de cor/etnia foi bastante diferente em nossas coortes do sétimo dia (Tabela 4S - Material suplementar). Isso pode ser devido ao uso comum do rótulo "outras" para descrever a cor/etnia do paciente. Estudos futuros que caracterizem os dados demográficos nesses conjuntos de dados podem ajudar a garantir que a pesquisa nessa área seja representativa dos pacientes que assistimos. Optamos por incluir apenas os pacientes que estavam na UTI por pelo menos 7 dias, em vez de avaliar os pacientes que estavam vivos no primeiro e no sétimo dia, para minimizar o risco de viés de tempo imortal. Apresentamos uma avaliação dos pacientes que saíram da UTI antes do sétimo dia e mostramos que eles eram bem parecidos com suas respectivas coortes do sétimo dia em termos de idade, sexo, cor/etnia e ICC, mas tinham menos probabilidade de atender aos critérios Sepsis 3 (Tabelas 5S e 6S - Material Suplementar). Isso sugere que as coortes finais que usamos para análise tinham maior probabilidade de estar em estado crítico, o que limita a generalização de nossos resultados.

Nossa análise também foi limitada, pois avaliou a contribuição da disfunção orgânica no risco de mortalidade apenas no primeiro e no sétimo dia. Escolhemos essa estratégia para testar nossa hipótese de que o impacto da disfunção orgânica varia com o tempo. Entretanto, essa estratégia não nos permitiu considerar a interação de dois órgãos (por exemplo, na síndrome cardiorrenal ou no choque hepático) e a diversidade de tratamentos que os pacientes podem receber entre o primeiro e o sétimo dia. Intervenções como intubação, terapia de substituição renal ou oxigenação por membrana extracorpórea podem afetar a forma como o peso de cada componente do órgão varia ao longo do tempo. Outros trabalhos nessa área devem avaliar a contribuição de diferentes sistemas de órgãos no risco de mortalidade em vários cenários clínicos em uma resolução temporal maior.

## Data Availability

Os dados que sustentam os achados deste estudo estão disponíveis no MIMIC-IV (doi.org/10.1093/jamia/ocx084) e no eICU-CRD (doi.org/10.1038/sdata.2018.178). Ambos os conjuntos de dados estão disponíveis publicamente por meio da PhysioNet (http://physionet.org).
